# Financing and collaboration on research and development for nodding syndrome

**DOI:** 10.1186/s12961-016-0091-6

**Published:** 2016-03-17

**Authors:** Christine Årdal, John-Arne Røttingen

**Affiliations:** Department of International Public Health, Norwegian Institute of Public Health, Oslo, Norway; Infection Control and Environmental Health, Norwegian Institute of Public Health, Oslo, Norway; Department of Health Management and Health Economics, Institute for Health and Society, University of Oslo, Oslo, Norway; Department of Global Health and Population, Harvard T.H. Chan School of Public Health, Boston, Massachusetts USA

**Keywords:** Market failures, Nodding syndrome, Neglected tropical diseases, Open knowledge innovation, Open science, Open source drug discovery, Open source innovation

## Abstract

**Background:**

Nodding syndrome is a neurological disease with no known cure or treatment, impacting children aged 3–18 years old, mainly in East Africa. Children progressively develop varying degrees of cognitive impairment which may lead to severe wasting, a vegetative state and, eventually, death. Despite its 50-year existence, little is known about its cause, risk factors and prognosis. It is a disease where markets will not provide solutions because the patients are both too few and too poor, making it especially neglected. Open source innovation has been recommended as an approach to neglected disease research in order to maximize available funding through greater collaboration and openness to results. Nodding syndrome is a useful case to examine the relevance of open source innovation.

**Methods:**

We assessed the magnitude of research related to nodding syndrome, its availability, financing and the amount of collaboration. We surveyed researchers regarding their motivations, attitudes toward open source innovation concepts and barriers to greater collaboration.

**Results:**

Little research is occurring for nodding syndrome, but it is openly available and researchers are highly collaborative. The disease is largely unknown, which is partly attributed to WHO not classifying nodding syndrome as a neglected tropical disease and not including it in any formal programme. Impacted countries, particularly Uganda, demonstrate a strong degree of ownership through both authorship and research financing. Nodding syndrome researchers have been allocated a total of €5 million from 2013 to 2019 in grant funding. Annual financing, due to three new grants, doubled from 2014 to 2015.

**Conclusions:**

Nodding syndrome, a disease previously ignored by the international community, is starting to receive greater attention, although financing remains modest. If infectious, a larger epidemic could take the world by surprise. Open source innovation can likely help by sharing research protocols (to avoid duplication) and early research results (to adjust to the findings of others). The existing scientists have already endorsed open source innovation, but increased financing is needed. The support of just a few high-income countries could reap a large impact.

## Background

Nodding syndrome (NS) is a neurological disease of unknown pathogenesis occurring primarily in East Africa, where a previously healthy child, typically aged between 3 and 18 years, experiences head nodding episodes potentially combined with epileptic seizures [1-3]. The episodes may be triggered by cold temperatures or food [4,5]. During the episodes children lose contact with their surroundings, which can be fatal if close to a cooking fire or deep water source. Some parents have resorted to protecting their children by tying them to trees [6]. Children progressively develop varying degrees of cognitive impairment which may lead to severe wasting, a vegetative state and, eventually, death [1,4,7]. There is no known cure or agreed treatment regimen despite the disease having been recognized for more than 50 years [5,8,9]. It is unknown if NS is infectious (it is clustered within families and regions [4]), genetic, related to onchocerciasis (most affected children are infected with *Onchocerca volvulus*) or measles, war, malnourishment, fungal contamination of food, or a combination of these factors [1,9]. Children are treated symptomatically with antiepileptic drugs, with unclear benefit [4]. Due to the unknown origin of the disease, patients and caregivers are often stigmatized [6].

The disease is contained to three low-income countries (South Sudan, Tanzania and Uganda), although it may also be present in other onchocerciasis-endemic areas [5,10]. Additionally, it impacts relatively few children (i.e. tens of thousands), with the actual prevalence difficult to ascertain since there is no formal reporting of cases. In northern Uganda, between 2012 to 2013, during which time there was an epidemic of nodding syndrome, there were an estimated 6.8 probable cases of NS per 1,000 children [11]. By contrast, in Tanzania, NS appears to be stable and endemic [2].

During 2012–2013, WHO convened three separate initiatives important to NS. Firstly, with the epidemic of NS in Uganda creating a greater focus on the disease, WHO, in collaboration with the Ugandan Ministry of Health and the United States’ (US) Centers for Disease Control and Prevention (CDC), convened the first scientific meeting on NS in Kampala in 2012 [12]. A formal definition of the disease was devised, and a collaborative research framework was agreed. WHO was tasked with coordinating collaborative research, including updating stakeholders on research planning, implementation and emerging results.

Secondly, in 2013, WHO published its ‘2020 Roadmap on NTDs’ [13] focused on 17 priority, neglected tropical diseases, but did not include NS. Rather, NS was classified on WHO’s website as one of seven “*other neglected conditions*” and not displayed on the same webpage as the 17 priority neglected tropical diseases.

Finally, WHO’s Consultative Expert Working Group on Research and Development: Financing and Coordination, delivered its report in 2012 [14], issuing recommendations on how to increase research and development (R&D) of diseases that largely impact developing countries, i.e. diseases like nodding syndrome. One of its recommendations was to encourage open source innovation, that is, the sharing of research results and innovations freely without legal or contractual restrictions or payment in a collaborative, typically virtual environment [14]. The rationale behind open source innovation is that greater collaboration will lead to cost savings in the form of less administration and avoidance of duplicative research as well as the creation of a dynamic collaboration where new ideas and perspectives can be heard [15, 16].

NS is a textbook example of where markets fail to provide solutions due to insufficient profit potential. In the case of nodding syndrome, there are not only too few patients, but they are also located only in low-income countries. This is a case where open source innovation could be advantageous in order to maximize the R&D financing available.

In this paper, we evaluate NS R&D against ‘open’ R&D approaches, by examining the following questions:

- Are NS research results (both data and biomedical samples) openly available?
- Is NS research performed collaboratively and what are scientists’ and organizations’ motivations for participation?
- How much financing does NS research receive?
- What are the barriers to greater research collaboration?

## Methods

In an attempt to identify all NS research (ongoing and complete), we examined publications, clinical trials and patents. On February 6, 2015, we extracted all research articles from PubMed that contained either ‘nodding syndrome’ or ‘nodding disease’ (*n* = 34). Since so few articles were retrieved, a Google Scholar search for ‘nodding syndrome’ was performed on May 5, 2015, with many more results (*n* = 176), including news items, citations, conference announcements as well as unrelated research. After reviewing these items, the relevant research articles were mostly duplicates (*n* = 33) with 16 additions. From these cumulative articles (*n* = 50), we extracted data about the co-authors, the funding sources and whether the article was available open access.

Corresponding authors (*n* = 26) were asked to complete an online survey (see Appendix) which included the researcher’s motivations for performing NS research, his/her employer’s perceived motivation, use of patents, availability of biological samples as well as perceptions regarding open source innovation concepts. In the invitation to participate in this survey, respondents were assured that all survey results would be held strictly confidential; that participation was completely voluntary and had no impact on any interaction with the Norwegian Institute of Public Health or the Norwegian government; and that the final article would be shared with respondents and published in an open access journal. A 62% (*n* = 16) survey response rate was achieved. Two follow-up questions were sent via e-mail. The first was sent directly to the 16 respondents asking for greater elaboration regarding barriers to NS research; nine responded. The second follow-up question was sent in February 2016 to all invited participants of the Gulu 2015 conference (*n* = 32), asking researchers to identify their financing sources and amounts; 11 responses were received.

On February 26, 2015, we queried WHO’s International Clinical Trials Registry Platform (http://apps.who.int/trialsearch/) for all clinical trials related to the condition ‘nodding syndrome’ (*n* = 1). On February 26, 2015, we searched the World Intellectual Property Organization’s Patentscope (https://patentscope.wipo.int) for patents which contained the word ‘nodding syndrome’ and/or ‘nodding disease’; no patents were identified.

To determine funding patterns of grant recipients of neglected disease R&D, we classified those institutions receiving financing in 2013 as reported by G-FINDER [17] as either high-, medium- or low-income utilizing World Bank country income classifications.

We sought approval for our research portfolio from the Norwegian Committees for Medical and Health Research. The Committee decided that our research did not require their ethical approval since we were studying collaboration amongst scientists and not patients.

## Results

### Open research

There is very little research in total about NS. Only 50 research articles were found, the oldest article being from 2008. This is likely due, in part, to no common name being determined for the disease prior to 2012. However, searches for ‘nodding disease’ did not increase the number of articles retrieved. This, combined with the registry of only one clinical trial (a randomized trial of oral pyridoxine and conventional anti-epileptic therapy initiated in 2012 and financed by the CDC in collaboration with the Ugandan Ministry of Health [18]), points to the reality that little research is occurring for NS.

Encouragingly, though, the published research that does exist is open access (86%; *n* = 44), meaning that it can be read online without charge. Overall, 63% of survey respondents collected biological samples and 60% of these are available to other researchers. Not surprisingly, given the little to no market potential, no patents related to NS were identified and survey respondents confirmed that there are no future patent plans.

### Collaboration

The 50 articles were exclusively written by public sector individuals (by academicians, governmental employees or private citizens). Authorship of the research articles was highly concentrated. Five corresponding authors wrote almost half (*n* = 23) of all of the research articles. Three of these authors gave a Ugandan affiliation (although one is German). Overall, 75% (*n* = 38) of the articles included at least one author from South Sudan, Tanzania or Uganda. Nine articles were solely authored by impacted country researchers.

The articles demonstrated significant collaboration. Over half (*n* = 31) of the articles included authors from more than one continent. Six articles included a co-author from WHO. Almost all respondents found their collaborating researchers through personal or professional networks.

In total, 81% of respondents were still performing research for NS at the time of the survey and 75% planned to continue. They were motivated by the intellectual stimulation of NS research (75%), improving the world to find a cure (69%), and networking with fellow researchers (63%). Employers were thought to be motivated by their mandate to advance knowledge creation (83%).

When asked about utilizing a research-sharing online platform, respondents were positive (75%) about placing their ongoing summarized research results or working papers there, with almost unanimity (92%) stating that the main benefit would be access to early research results. There was also near consensus amongst survey respondents that such a platform would help them to identify research gaps (81%), become aware of other ongoing research projects (75%) and reduce any potential duplication of efforts (75%).

One of the recommendations from the 2012 WHO meeting was for WHO to establish a Nodding Syndrome Research Coordination Group including a mechanism to ensure that this type of registry data is shared. Nevertheless, we found no evidence that WHO has made any progress on these recommendations. However, independently, two articles were published in 2015 outlining the research needs for NS [1,5]. Additionally, a scientific meeting focused solely on NS occurred in Uganda in July 2015 [9]. This meeting, convened by Uganda’s Gulu University, gathered 80 participants from four continents to present and discuss their research results on NS [9].

### Financing

In 2015, NS received approximately €1.3 million in financing, solely from public sector bodies, including the European Union, the Dutch government and the US National Institutes of Health. This is a substantial increase from previous amounts, with approximately €580,000 in 2014 and under €100,000 in previous years. At least six authors stated that they had not received any funding for their NS research prior to 2014. Financing is expected to remain relatively stable, with €1.2 million in 2016 and €1 million in 2017. In total NS will receive at least €5 million in financing from 2013 to 2019.

### Barriers

Survey respondents repeatedly mentioned that obtaining financing is one of the largest barriers to performing NS research. They expressed the main difficulties in attracting funding is that NS is not considered a funding priority or a public health threat as well as that the disease is simply unknown. They asserted that WHO facilitates this unawareness by not including it in WHO’s formal programme for neglected tropical diseases.

That 10 out of the 26 unique corresponding authors represent institutions located either in Uganda or South Sudan may also contribute to the difficulties of securing R&D funding. In an analysis of G-FINDER data for 2013, we found that 81% of grant recipients focusing on basic research for neglected diseases (*n* = 1,049) are located in high-income countries as opposed to 2% (*n* = 29) in low-income countries (utilizing World Bank country income classifications). If viewed in terms of funding allocations, 85% (USD 650m) of basic research financing is paid out to high-income country recipients as opposed to 1% (USD 9.7m) to those in low-income countries (Table 1) [19]. Of course, this may be misleading as low-income country recipients are often subcontractors to high-income country institutions.

**Table 1:** Recipients for basic research for neglected disease grants in 2013 [19] using World Bank income classifications

Income group	Number of grants (%)	Sum of grants (%)
Low	29 (2%)	USD 9,729,342 (1%)
Lower middle	62 (5%)	USD 19,329,460 (3%)
Upper middle	118 (9%)	USD 33,397,384 (4%)
High	1049 (81%)	USD 649,722,068 (85%)

## Discussion

The results of this paper demonstrate that NS research is being performed in an open, collaborative way with the main barrier being the lack of financing. However, there are a number of limitations to this study. Firstly, the financing amounts may be understated since two important financers did not respond to our query. There may be additional research articles where NS is identified as another disease since the official name was only agreed upon in 2012. Since the number of researchers identified for NS is small, our dataset also represents a small sample size. We also did not probe into any concerns that researchers may have regarding collaborative models and data-sharing. However, we believe that, despite these limitations, our findings have merit.

The premise of open source innovation is that freely sharing research results and innovations without legal restrictions or payment will encourage greater collaboration, save administration costs and avoid duplication of efforts. This is especially relevant for a disease like NS, with relatively small numbers of affected children located in some of the poorest countries of the world. It is imperative to avoid duplication of research on this vulnerable population.

Despite its historical connections with the software industry, an open source approach does not necessarily need to be technical. Rather, an open approach focuses on access to early research results and the creation of a collaborative environment. The primary NS researchers have already agreed to these principles at their 2012 scientific meeting, and the majority reconfirmed their willingness in our survey. They are living up to their pledges by ensuring transparency through open access publishing and sharing preliminary research results at the 2015 scientific meeting [9]. Nevertheless, a greater number of researchers is likely needed to find solutions.

Collaboration on basic research is needed by epidemiologists, entomologists, ecologists, hydrologists, experts in onchocerciasis vector control, clinicians, anthropologists, public health experts, national and local authorities, and the local population itself [1]. This increased collaboration cannot be achieved if NS remains unknown, the principle barrier given to greater research collaboration. WHO may have recently assisted in this regard. In 2015, NS was classified on WHO’s website as one of seven “*other neglected conditions*”, and it was not displayed on the same webpage as the 17 priority neglected tropical diseases. In 2016, it is now classified by WHO as an emerging disease under emergencies preparedness and response [20], although it is not included in WHO’s list of top emerging diseases likely to cause major epidemics [21]. An inclusion in the list of emerging diseases with potential epidemic ramifications will likely gain greater attention to NS. Additionally, the large increases in financing should generate more research, which will also add to the public awareness of the disease.

Financing for NS is still relatively modest though, with annual financing of about €1 million. Comparatively, in 2013, Buruli ulcers (with about 6,000 reported annual cases [22]) received USD 7 million for R&D and human African trypanosomiasis (sleeping sickness; with about 7,000 reported annual cases [23]) received USD 39 million [17]. G-FINDER, an annual report that tracks R&D financing for neglected diseases, should also be encouraged to monitor the R&D financing devoted to NS. This would also improve awareness of the disease.

Impacted countries, particularly Uganda, demonstrate a strong degree of ownership through both authorship and research financing. This may contribute to insufficient research financing since donors are more likely to grant funds for neglected disease research to high-income country recipients.

## Conclusions

It is unlikely that greater collaboration can be achieved without greater financing. Open access journals and participation in scientific meetings are generally not free. Today, NS research is underfunded to the extent that it relies upon researchers using their own personal funds. This is neither sustainable nor the path to finding a cure. Ideally, several high-income governments would champion the disease. This is an opportunity where a relatively modest investment could reap a large impact. Uganda is already demonstrating significant country ownership here. High-income countries could pledge a matching grant, for example, on a scale of 10 times investment for every dollar invested by an impacted country.

There will likely never be a viable business model to find a cure for NS. The costs of developing a vaccine, new medicine or diagnostic, or eradicating a parasite are large [24,25]. There is little to no revenue to be had, unless it is perceived as a global threat. Solving this problem, therefore, is the responsibility of governments (both impacted countries and donors) as a global public good. Maintaining the current level of openness and collaboration (but increasing the scale of researchers) should make these public investments efficient by focusing them on the research and avoiding administrative costs, like patenting and contracting.

The Ebola and Zika virus crises have awakened the global community to seemingly small, rural, public health threats. So much is unknown about NS that new epidemics, like the one in Uganda from 2006 to 2013, could take countries and the world by surprise. Most brutally, the current apathy toward NS is slowly destroying the lives of previously healthy children while forcing their families to stand helplessly by and watch.

## Appendix

### Survey to nodding syndrome corresponding authors

1. Do you still perform research related to nodding syndrome?
  - Yes
  - No
  - I have never performed research on nodding syndrome (Go to end)
2. How many years have you performed research on nodding syndrome?
3. Do you plan to continue researching nodding syndrome?
  - Yes
  - No
  - I don’t know
4. What is your personal motivation for performing research on nodding syndrome? (Please check all that apply)
  - I find research on nodding syndrome intellectually stimulating and scientifically interesting
  - I believe that performing nodding syndrome research will assist the progression of my career
  - I am interested in being a part of a network of nodding syndrome researchers
  - My family/friends are at risk from nodding syndrome; I want to make a difference in their lives
  - I want to improve the world by doing my part to find a cure for nodding syndrome
  - I did not select nodding syndrome as my field of research – my employer has as a part of my duties
  - I perform nodding syndrome research because that was the funding that I was able to secure
  - Other (free text field)
5. What type of organization is your employer? (Please select the one that best applies)
  - A university or college
  - A government research institute
  - A for-profit company (for example, a pharmaceutical company)
  - A private non-profit research institute (for example, foundation-based)
  - I am self-employed (Go to Question 7)
  - I am unemployed (Go to Question 7)
  - I am retired (Go to Question 7)
  - I am a student (Go to Question 7)
  - Other (free text field)
6. What do you believe is your employer’s motivation for performing nodding syndrome research? (Please check all that apply)
  - My employer is a publicly-funded institution with a mandate to advance knowledge creation
  - My employer believes that there is a potential profit in researching nodding syndrome
  - My employer believes that it has a social responsibility to research nodding syndrome in order to improve health in low-income countries
  - There is external funding readily available to perform nodding syndrome research
  - My employer educates students regarding neglected diseases and therefore my research assists in the students’ formal education
  - My employer is located in a nodding syndrome-endemic country, and it is a national priority to research nodding syndrome
  - My employer leaves my field of research up to my own discretion
  - My nodding syndrome research activities are not a part of my paid job
  - Other
  - I don’t know
7. Have you or your organization applied for any patents on your nodding syndrome research, or plan to apply for patents on your nodding syndrome research?
  - Yes (Go to Question 8)
  - No (Go to Question 10)
  - I/we have not yet decided (Go to Question 8)
  - I do not know (Go to Question 10)
  - My research is not patentable (Go to Question 10)
8. What stage would you designate the research that you have patented or plan to patent? (Please select the one that best applies)
  - Basic research (i.e. research into the mechanisms/organisms that cause nodding syndrome)
  - Target identification and validation
  - Finding and optimizing lead compounds
  - Developing processes for making candidate drugs, vaccines or diagnostics
  - Clinical trials
  - Other
  - None of the above
  - I don’t know
9. Why did you or will you patent your research results on nodding syndrome? (free text)
10. Did your research collect any biological samples (i.e. blood specimens, etc.)?
  - Yes (Go to Question 11)
  - No (Go to Question 12)
  - I don’t know (Go to Question 12)
11. Have you made these specimens available to external researchers, for example, through a biobank or other repository?
  - Yes
  - No
  - I don’t know
12. Did your research generate any chemical or molecular compounds?
  - Yes (Go to Question 13)
  - No (Go to Question 14)
  - I don’t know (Go to Question 14)
13. Have you made these compounds available to external researchers, for example, through a library or other repository?
  - Yes
  - No
  - I don’t know
14. As identified through PubMed, only 29 research articles have ever been published on nodding syndrome or nodding disease. How did you find your collaborating partners? (free text)
15. Since there are so few researchers focused on nodding syndrome, do you believe that master degree students could be one potential source of additional research capacity? Please describe why or why not and if so, which academic fields would be particularly valuable (free text)
16. One area that we are considering is the helpfulness of registries of previous and ongoing research, particularly related to neglected diseases. The idea is that research projects would be registered once they are funded or started. Therefore, in theory, you could be alerted to specific, newly initiated research projects in a topic of your choice.
  What could be the benefits of a registry covering all of nodding syndrome research? (Please check all that apply)
  - The registry could help me to become aware of related research projects
  - The registry could help me to identify gaps within my field of research
  - The registry could help me to determine my future research projects
  - The registry could help me to identify potential collaborators for my research
  - The registry could reduce duplication of research
  - Other (free text field)
  - I do not see any benefits of such a registry
  - I don’t know
17. We are also examining the potential of placing preliminary research results on a publicly-available website for comment or review. Prominent journals have indicated that sharing research results in this fashion is acceptable and do not preclude the research from later publication.
  Would you consider sharing your preliminary research results on nodding syndrome on a publicly-available website?
  - Yes (Go to Question 18)
  - No (Go to Question 20)
  - I don’t know (Go to Question 20)
18. What types of results might you be willing to share? (Please check all that apply)
  - Raw results data
  - Summarized results data
  - Working papers in development
  - Other (free text field)
  - I don’t know
19. What would be the benefit of sharing research results? (Please check all that apply)
  - Giving access to early research results
  - Commenting on others’ research
  - Establishing research collaboration
  - Other (free text field)
  - I don’t know
20. Do you have additional ideas on how research on nodding syndrome can be strengthened internationally, e.g. through collaboration and coordination, financing, or other means? (free text)
21. Do you have any additional comments? (Free text)
